# Electroacupuncture Pretreatment Regulates Apoptosis of Myocardial Ischemia-Reperfusion Injury in Rats Through RhoA/p38MAPK Pathway Mediated by miR-133a-5p

**DOI:** 10.1155/2021/8827891

**Published:** 2021-03-08

**Authors:** Yongli Han, Song Chen, Hua Wang, Xing-ming Peng

**Affiliations:** ^1^College of Acupuncture and Orthopedics, Hubei University of Traditional Chinese Medicine, Wuhan, Hubei 430061, China; ^2^Hubei Collaborative Innovation Center for Acupuncture Treatment, Wuhan, Hubei 430061, China; ^3^Second People's Hospital of Jingzhou, Jingzhou, Hubei 434099, China; ^4^Institute of Internal Medicine, Huangjiahu Hospital of Hubei University of Traditional Chinese Medicine, Wuhan, Hubei 430065, China

## Abstract

The electroacupuncture (EA) pretreatment possesses a beneficial effect on myocardial ischemia/reperfusion (I/R) injury. However, the molecular mechanism of the EA effect is not fully understood. The study aimed to explore the protective effect of EA pretreatment on myocardial ischemia-reperfusion injury (MIRI) and apoptosis-related mechanisms in rats. Rats underwent in vivo myocardial ischemia-reperfusion, EA pretreatment, or intravenous injection of antagomirs. Cardiac function, infarct area, and myocardial cell apoptosis were measured. Meanwhile, the expressions of MKK3, MKK6, p38MAPK, Bax, Bcl-2, and Caspase-3 were also detected. We found that EA pretreatment significantly reduced infarct area and myocarpal cell apoptosis and enhanced cardiac function. EA pretreatment decreased the expression of Bax, Caspase-3, MKK3, MKK6, p38MAPK, Bax, and Caspase-3. In conclusion, The EA pretreatment down regulated the expression of MKK3, MKK6, and p38MAPK through the RhoA/p38MAPK pathway. EA pretreatment protect MIRI rats from apoptosis by down regulating the expression of MKK3, MKK6, and p38MAPK, thereby reducing the expression of Bax, Caspase-3 and up regulating the expression of Bcl-2, which mechanism is closely related to the RhoA/p38MAPK pathway mediated by miR-133a-5p.

## 1. Introduction

Acute myocardial infarction (AMI) and subsequent heart failure cause significant morbidity and mortality worldwide [1]. Although early coronary reperfusion after AMI is a typical treatment method, the accompanying myocardial ischemia-reperfusion injury (MIRI) and related cardioprotective effects and mechanisms remain unresolved [2, 3]. MIRI causes myocardial cell necrosis, apoptosis, arrhythmia, and other cardiac dysfunctions [4]. Our previous research showed that electroacupuncture (EA) at “Neiguan” (PC6) participates in the regulation and reduction of the oxidative stress response, inflammatory response, calcium overload, and apoptosis during reperfusion injury via the p38MAPK signal pathway [5, 6]. In addition, p38MAPK is a key factor in myocardial apoptosis signal transduction after ischemia-reperfusion (I/R). In the myocardial ischemia-reperfusion (MIR) rat model, EA pretreatment at PC6 inhibited p38 phosphorylation expression and reduced MIR of apoptotic cells in rats, thereby improving its cardiac function and protecting it from MIR [7, 8].

Recently, the RhoA/p38MAPK pathway gradually gained interest in the field of apoptosis. Studies have shown the role of RhoA or RhoA effector molecule, Rho kinase (ROCK), in protecting the myocardium from reperfusion injury after myocardial ischemia, related to myocardial cell survival and Caspase-3 activation [9]. *In-vitro* studies have found that the RhoA/ROCK pathway mediated high glucose- (HG-) induced apoptosis of cardiomyocytes via oxidative stress and activation of p38MAPK and JNK. The p38MAPK [10] inhibitor effectively inhibited early glucose-induced cardiomyocyte apoptosis via RhoA, reduced Bax mRNA expression, Bax/Bcl-2 protein expression ratio, and Caspase-3 protein level and upregulated Bcl-2 mRNA expression, thus indicating the indispensable regulatory role of the RhoA/p38MAPK pathway in apoptosis [11].

The EA pretreatment of “point matching of specimen” (Biao Ben Pei Xue) refers to the acupoints that fix the positive qi and eliminate the evil qi as the standard, and the combination acupoints method that uses both PC6 and “Zusanli” (ST36) together. Our previous studies confirmed [12] that miR-133a-5p expression in rats significantly decreased after myocardial ischemia. The miR-133a-5p expression further significantly inhibited or increased after injection of inhibitors or agonists. A close regulatory relationship was found between miR-133a-5p and Caspase-3. The EA pretreatment of “point matching of specimen” effectively enhanced the expression of miR-133a-5p and had a more significant regulatory effect than the EA pretreatment at PC6. In this study, EA was used to pretreat PC6 and ST36 to explore its role in MIRI apoptosis via the miR-133a-5p-mediated RhoA/p38MAPK mechanism.

## 2. Materials and Method

### 2.1. Ethical Approval

This experiment was carried out per the requirements of the document “Guiding Opinions on Treating Experimental Animals,” no. 398 of the Ministry of Science and Technology, reviewed and approved by the Ethical Review Committee of the Laboratory Animal Center of the Hubei Academy of Medical Sciences (Approval number: SXDX2019050 A). Significant efforts were made to minimize the animal number and reduce their suffering.

### 2.2. Instrument and Reagents

Homogenizer (Servicebio, China), desktop high-speed refrigerated microcentrifuge (DragonLab, Spain), fluorescence quantitative PCR instrument (ABI), ultramicro spectrophotometer (Thermo Scientific NanoDrop2000, US), and standard reagent-type pure water meter (Qingdao Fulham Technology Co., Ltd., China) were used. Electrophoresis apparatus (Beijing Liuyi Instrument Factory, China), transfer electrophoresis tank (Beijing Liuyi Instrument Factory), microplate reader (Ditek), refrigerated centrifuge (Hunan Xiangyi Laboratory Instrument Development Co., Ltd., China), decolorization shaker (Beijing Liuyi Instrument Factory), scanner (Canon), and inverted fluorescence microscope (Nikon) were used. Anti-rabbit/mouse universal immunohistochemical kit REAL ™ EnVision+/HRP Rabbit/Mouse was used (Dako Denmark A/S, Denmark). DAPI (Servicebio), *in-situ* apoptosis kit (Roche, Switzerland), DMEM/H medium (Thermo Scientific HyClone), Opti-MEM media (Gibco™, US). RNA extraction solution (Servicebio), chloroform (Sinopharm Group Chemical Reagent Co., Ltd., China), HyPure^TM^ Molecular Biology Grade Water (HyClone), RevertAid First Strand cDNA Synthesis Kit (Thermo Scientific), and FastStart Universal SYBR Green Master (Rox) (Servicebio) were used. SDS-PAGE gel preparation kit (ASPEN), RIPA total protein lysate (ASPEN), BCA protein concentration determination kit (ASPEN), Protease Inhibitor Cocktail (Roche), Protein Marker (Thermo Scientific), and 0.45 *µ*m PVDF membrane (Millipore) were also used.

### 2.3. Experimental Animals

Fifty specific pathogen-free (SPF) male Wistar rats weighing 180–220 g were procured from the Laboratory Animal Center of Hubei Academy of Medical Sciences, China (Laboratory Animal Facility Certificate no. 00133292). Animals were fed in a standard animal room and adaptively fed for one week at the experiment's start.

### 2.4. I/R Model

In this study, a MIR model was created in rats using physical ligation and push tube method [13]. Rats were anesthetized, fixed on a rat table, and monitored by ECG. The skin of the rat's neck was cut open, the trachea exposed a “T”-shaped incision made at the tracheal cartilage ring, and the catheter was inserted and connected to the ventilator (f 50 times/min, I/E 1:1.5, p-limit of 20 cm H_2_O). The skin was cut through the third and fourth intercostal incisions of the left sternum, the muscle layers, and the pleura bluntly separated with hemostatic forceps and opened. The pericardial capsule was cut to expose the heart, and a 2 × 6 round needle with no. 6–0 medical nylon monofilament thread at 2∼3 mm below the left atrial appendage bypassed the anterior descending coronary artery and drawn out from the paracone groove of the pulmonary artery. A pressure equalizer polyethylene (PE) tube was placed on the silk thread, and a slip knot was used to create an ischemia model. The left ventricular anterior wall swells outwards, and ST-segment elevation in ECG is the sign of ischemia. During reperfusion, the purple color of the left ventricle anterior wall ischemic area disappears, the elevated ST-segment decreases by more than 1/2, and local reactive hyperemia occurs. Inflammatory edema and exudation, that is, the I/R model, were successfully prepared.

### 2.5. Pretreatment Methods

All animals were numbered, and after one week of adaptive feeding, the random number table was checked and divided into five groups (*n* = 10). Sham operation group (Sham): bundled once a day for seven days, 20 min/time; the heart exposed after the thoracotomy on the 8th day without other treatment. I/R group: bundled once a day for seven days, 20 min/time; on the 8th day after thoracotomy, 20 min of ischemia and 40 min of reperfusion were administered. EA pretreatment group: after wearing the self-made rat clothes, acupoints were selected for the rats [14], and PC6 was taken between the ulnar and radial joints about 3 mm away from the wrist joint on the inner side of the forelimb. The puncture depth was about 5 mm. At the postero-lateral side of the knee joint, 5 mm below the fibular head, bilateral ST36 was taken to the left and right with an acupuncture depth of about 2 mm. PC6 and ST36 were connected to the EA treatment instrument (HANS-200), using continuous wave, with 2 Hz frequency, 1 mA intensity and power on 20 min, and once a day for seven days; I/R was performed on the 8th day. miR-133a-5p inhibitor group (Antagomirs): bundled one time a day for seven days, 20 min/time; on the 5th day, the antagomirs (0.6 *µ*mol/L) were given continuously (80 mg/kg tail vein injection) three days before I/R; EA pretreatment + Antagomirs group: EA pretreatment for seven days; antagomirs were given on day 5, before I/R.

### 2.6. Echocardiographic Evaluation

According to the American Society of echocardiography guidelines, the mice from each group were measured in M-Mode using a high-resolution small animal ultrasound echocardiographic evaluation system after the treatment. The transducer was placed on the chest, and three independent cardiac cycle images of the left parasternal short-axis were observed. The left ventricular end-diastolic diameter (LVDd) and left ventricular end-systolic diameter (LVDs) in the images were measured. After conversion, the left ventricular ejection fraction (LVEF) and the left ventricular short-axis shortening rate (LVFS) were obtained for statistical analysis. After the determination, thoracotomy was performed, and the infarcted area was cut out for preservation.

### 2.7. Evans Blue and TTC Double Staining Assay

After 20 min of ischemia and 40 min of reperfusion, the thoracic cavity was opened. The left coronary artery of the original descending artery was religated, and 0.2 mL of 2% Evans Blue dye was injected into the right ventricle to stain the myocardium perfused by the corresponding coronary artery. The heart was removed from the body, the left ventricle was separated, and the heart was cut into 4 to 6 pieces from the heart's apex along the direction perpendicular to the heart's long axis. The slices were placed in 0.7 mL of 1% 2,3,5-triphenyltetrazolium chloride (TTC) phosphate buffer, incubated at 37°C for 15 min, and then both sides were photographed using a high-resolution dissecting microscope. After staining, the normal area was blue, the area-at-risk was light red, and the infarct area was white. Each area was statistically analyzed by Image J software (National Institutes of Health, US) to calculate the myocardial area-at-risk/left ventricular (AAR/LV) and infarct size/area-at-risk ratio (IA/AAR).

### 2.8. TUNEL Staining Assay

Paraffin sections of rat myocardial infarction tissues were placed in an oven at 65°C for 2 h, dewaxed in water, and washed three times with phosphate-buffered saline (PBS). The sections were placed in the EDTA buffer for microwave repair and power off after medium heat to boiling. Low heat to boiling stopped, and the sections were placed in a 3% hydrogen peroxide solution and incubated for 10 min at room temperature; the purpose is to block endogenous peroxidase. It was washed three times with PBS for 5 min each and blocked with 5% bovine serum albumin (BSA) for 20 min after drying. The BSA solution was removed, and a diluted primary antibody (50 *µ*L) was added to each section covering the tissue overnight at 4°C. The PBS solution was removed, and a secondary antibody of the corresponding species (50–100 *µ*L) was added to each section and incubated at 4°C for 50 min. After washing with PBS solution, a freshly prepared 3,3′-diaminobenzidine (DAB) solution (50–100 *µ*L) was added, and the color development was controlled under a microscope. After color development is complete, it was rinsed with distilled water and counterstained with hematoxylin and 1% hydrochloric acid-alcohol for differentiation. The section was further rinsed with distilled water, returned to ammonia, and rinsed with running water. The sections were subjected to gradient alcohol (70–100%) for 10 min, dehydrated, and dried, and transparent xylene was added and sealed with neutral gum. TUNEL-positive cells are characterized by brownish-yellow particles visible in the nucleus, and negative cells were without brownish-yellow particles. The apoptotic cardiomyocytes number was counted using Image J software (National Institutes of Health, US) , and the positive rate (%) of TUNEL-positive cells (positive cells/total cells *∗* 100) was calculated as the apoptosis index.

### 2.9. Immunohistochemistry Assay

Myocardial tissue was fixed with 10% paraformaldehyde, dehydrated, and embedded in paraffin, and conventional sections were 4 *µ*m. An anti-rabbit/mouse universal immunohistochemistry kit was used to process paraffin sections according to standard procedures. Reagent 1 (TdT) and reagent 2 (fluorescein-labeled dUTP) mixed solution (1 : 9, freshly prepared) (50–100 *µ*L) was added to the cells and incubated. Then, 2-(4-amidinophenyl)-6-indolecarbamidine dihydrochloride (DAPI) (50–100 *µ*L) was added and incubated at room temperature in the dark for 10 min and washed thrice in PBS in the dark for 5 min each. The slides were mounted with an antifluorescence quencher, and apoptotic cells were counted randomly in each section under six high-power field (×400) fluorescence microscopes. Image-Pro Plus 6.0 Image Analysis System (Media Cybernetics,US) was used to perform optical density analysis and record the integrated optical density (IOD) value.

### 2.10. RT-PCR Assay

Trizol reagent (1 mL) was added to the homogenate tube to precool, and then 100 mg of myocardial tissue from the infarct area was added. After homogenization, chloroform (250 *µ*L) and 0.8 volume of iso-propanol were added to the supernatant, inverted, mixed, and centrifuged to extract the RNA. RNase-free water (15 *µ*L) was added to the centrifuge tube to dissolve the RNA. The RNA concentration and purity were detected using Nanodrop 2000 to a final concentration of 100–500 ng/*µ*L. RNA solution (10 *µ*L) and 1 *µ*L oligo (dT) 18 were added to the PCR tube and made up to 50% with deionized water without ribonuclease (12 *µ*L). After incubation and cooling, 4 *µ*L 5×reaction buffer, 2 *µ*L 10 mM dNTP mix, 1 *µ*L RiboLock RNAase inhibitor (20 U/*µ*L), and 1 *µ*L RevertAi M-MuLV reverse transcriptase (200 U/*µ*L) were added and mixed with a gun. The reaction mixture was incubated in a PCR machine at 42°C for 60 min and end at 80°C for 5 min to inactivate reverse transcriptase.

The names and sequences of the synthesized primers are R-GAPDH-S: CTGGAGA A ACCTGCCAAGTATG, R-GAPDH-A: GGTGGAAGAATGGGAGTTGCT; R-P 38 (rz)-S: ACCACGACCCTGATGATGAGC, R-P38 (rz) –A: TAGGTCAGGCTCT TCCATCL2; S: TTGTGGCCT-TCTTTGAGTTCG, R-BCL2-A: GCATCCCAGCC TCCGTTAT; R-Casp3-S: GAAAGCCGAAACTCTTCATCAT, R-Casp3-A: ATGC CATATCATCGTCAGTTCC; R-BAX-S: TGAACTGGACAACAACATGGAG, R-B AX-AAGAGAAGAGAAGAGA-AGGAAAA: ACAGCTCCCAGCAGACCAGTT, R-MKK3-A: GCAGCAATGTCCG-TCTTCTTAGTT; R-MKK6-S: GGCTCCTGAA CGAATAAATCCA, R-MKK6-A: ACGGCTCTTCTACCACCTGTTT.

The following reaction system was prepared for each reverse transcription product in a 0.2 mL PCR tube in triplicate: 2×qPCR mix (12.5 *µ*L), 7.5 *µ*M gene primer (2.0 *µ*L), reverse transcription product (2.5 *µ*L), and ddH_2_O (8.0 *µ*L). After PCR amplification, a melting curve was obtained, and 2^–△△ Ct^ was used for result processing.

miRNA reaction buffer mix 2 x (10 *µ*L), buffer mix BSA, BSA RNA, BSA PrimeScript enzyme mix, and RNase free-H_2_O (5 *µ*L) were prepared in a 20 *µ*L reaction system. The reaction mixture was placed in the PCR instrument, with PolyA tail reaction at 42°C 30 min, reverse transcription reaction at 85°C, and inactivation of reverse transcriptase termination reaction under 85°C. The reaction mixture (20 *µ*L) containing PCR forward primer, 1 *µ*L qPCR primer, 2 *µ*L reverse transcriptase (RT), 10 *µ*L 2×SYBR premix Taq II, and 6 *µ*L dH_2_O were prepared. Then, 45 cycles were performed to obtain a melting curve. Using 2^–△△ Ct^ as an internal reference, relative quantification of the miR-1333a-5p level was carried out.

Primer: upstream, 5′-TCATATTTTGTTCCCCCCTTT-CAACC-3′; downstream, 5′-TATCGT TTGTTCTCTCCA-CTTCCTTCAC-3'. Primer: upstream, 5′-ATTGGA GCGAGATACAGAAGAGATT-3′; downstream, 5′-GGAGCGCTTTC AGAGATTTG-3'.

### 2.11. Western Blot Assay

Cell total protein extraction reagent was used to extract total protein of adherent cells, suspended cells, and tissues. The protein concentration of the sample was determined using a BCA protein concentration determination kit. The separation and concentrated gel were prepared separately, and the comb was pulled out and placed into the electrophoresis tank. The electrophoresis buffer was added, and the sample was added to the spotting well. Electrophoresis was performed at the constant voltage of 80 V for concentrated gel and 120 V for separation gel until bromophenol blue reached the gel plate's lower edge. The polyvinylidene fluoride (PVDF), Servicebio, China, membrane was activated with methanol, and the transfer membrane “sandwich” structure was placed in the direction of the positive and negative electrodes. The transferred membrane was added to the blocking solution and block at room temperature for 1 h. After removing the blocking solution, a diluted primary antibody was added and incubated at 4°C overnight. The diluted primary antibody was recovered, replaced with the diluted secondary antibody, and incubated at room temperature for 30 min. It was washed four times with TBST for 5 min each at room temperature on a shaker. The freshly prepared electro chemilumine scence (ECL) mixed solution was added dropwise to the membrane's protein side and exposed in a dark room. The film was scanned and archived, and the AlphaEaseFC Nanjing Changxiang Instrument Equipment Co., Ltd., China, software processing system analyzed the optical density value of the target band.

### 2.12. Statistical Methods

The experimental data were analyzed using SPSS 22.0 (IBM). Measurement data were described as *x* ± *s*, and a one-way analysis of variance using Tukey's test for multiple comparisons was used to assess differences between groups. The significance level was *P* < 0.05.

## 3. Result

### 3.1. Cardiac Function Assessment

**Figure 1:**
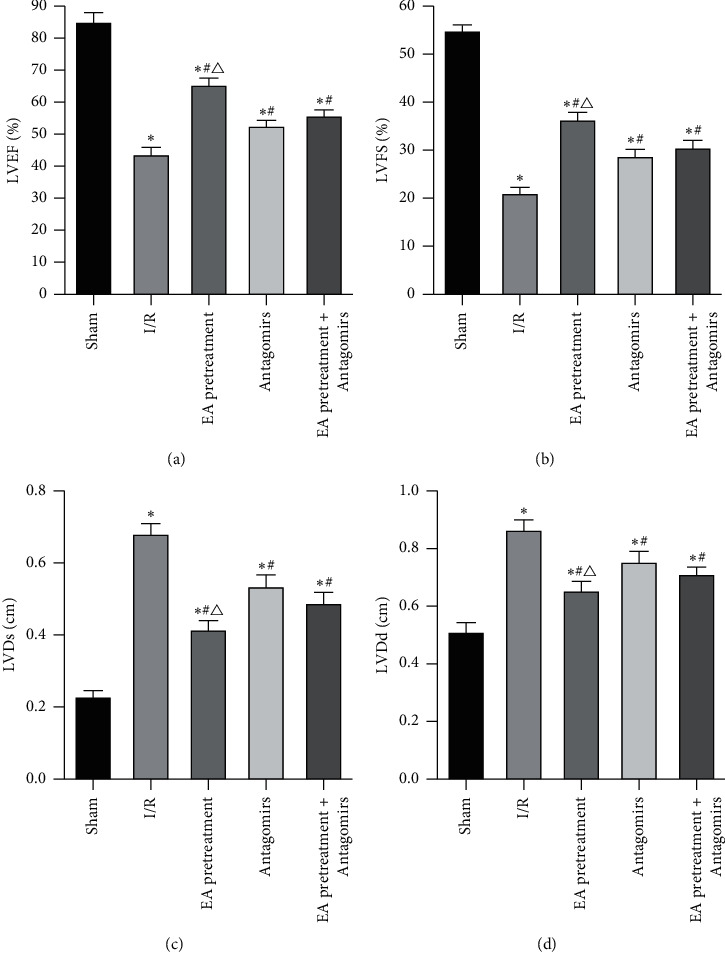
Comparison of LVEF (a), LVFS (b), LVDs (c), and LVDd (d) indexes of rat heart function in each group (*x* ± *s*, *n* = 10). ^*∗*^, *P* value <0.05 when compared to the Sham group; *P* value <0.05 when compared to the I/R group; *P* value<0.05 when compared to the Antagomirs and EA pretreatment + Antagomirs group.

EA pretreatment results on the cardiac function of MIRI in rats showed that all the indexes of cardiac function in the Sham group decreased at varying degrees. The effect of different interventions on the heart function of MIRI rats shows that compared with the sham operation group, the I/R group's various cardiac function indexes decreased to varying degrees; LVEF and LVFS were significantly decreased, and LVDs and LVDd were significantly increased (*P* < 0.05). Compared with the I/R group, the EA pretreatment group, EA pretreatment + Antagomirs group, and Antagomirs group all improved cardiac function in varying degrees. Among which the effect of the EA pretreatment group was the most significant (Figure 1).

### 3.2. Myocardial AAR/LV and IA/AAR Detection

**Figure 2:**
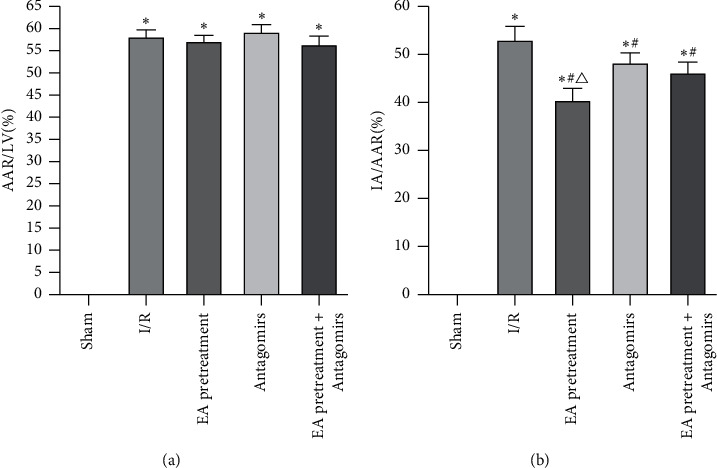
Comparison of AAR/LV and IA/AAR of rats in each group (*x* ± *s*, *n* = 10). ^*∗*^, *P*<0.05 compared to the Sham group; *P*<0.05 compared to the I/R group; *P*<0.05 compared to the Antagomirs and EA pretreatment + Antagomirs group.

**Figure 3:**
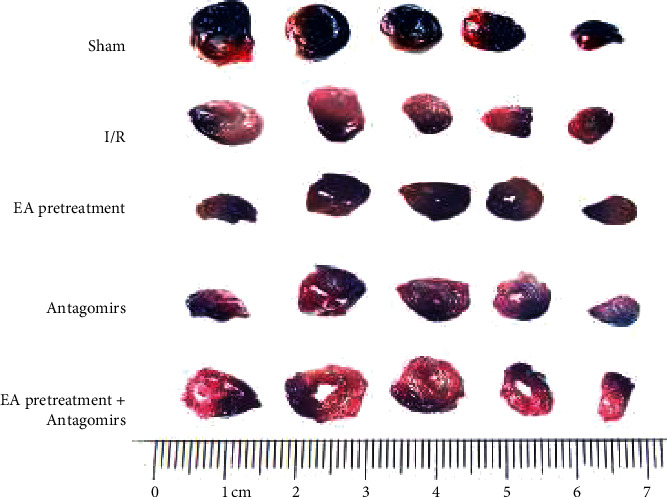
Typical myocardial staining of rats in each group. The blue area of Evans Blue staining is the normal area, the red part of TTC staining is ischemic myocardial tissue, and the pale part is the infarcted area. There was no obvious prominent white staining stained zone in rats of the Sham group; the white staining stained area increased in the I/R group. Compared with the I/R group, the white staining stained area decreased among the EA pretreatment group, EA pretreatment + Antagomirs group, and Antagomirs group, respectively.

EA pretreatment on myocardial ischemia and infarct size showed that no prominent white stained zone present in the Sham group rats, suggesting no infarction (Figures 2 and 3). Compared with the Sham group, the white stained area increased (*P* < 0.05) in the I/R group suggesting myocardial infarction. Compared with the I/R group, the white stained area decreased among the EA pretreatment group, EA pretreatment + Antagomirs group, and Antagomirs group. These results indicated that myocardial infarction improved significantly at various degrees, especially in the EA pretreatment group (*P* < 0.05).

### 3.3. Cardiomyocyte Apoptosis Detection

**Figure 4:**
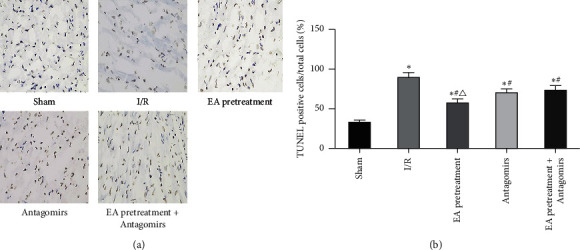
Effects of different treatments on rat cardiomyocyte apoptosis. (a) Apoptosis of myocardial tissue after TUNEL staining (original magnification×200); (b) myocardial apoptosis index (*x* ± *s*, *n* = 10). ^*∗*^, *P*<0.05 compared to the Sham group; *P*<0.05 compared to the I/R group; *P*<0.05 compared to the Antagomirs and EA pretreatment + Antagomirs group.

The apoptosis rate of myocardial cells in rats under EA pretreatment and antagonist conditions was compared to verify EA pretreatment role in myocardial apoptosis of MIRI in rats. No apoptotic cells were observed in the Sham group (Figure 4). Apoptotic cells were denser, and the apoptotic index increased significantly in the I/R group (*P* < 0.05). Apoptosis cells in the EA pretreatment group were significantly lower than the I/R group, and the cardiomyocyte apoptosis rate also decreased significantly (*P* < 0.05). Apoptotic cells slightly reduced in the Antagomirs group and the EA pretreatment group compared to the I/R group. Inhibition of apoptosis by EA pretreatment was attenuated by antagonist blocking (*P* < 0.05).

### 3.4. Positive IOD Values of Bax, Bcl-2, and Caspase-3

**Figure 5:**
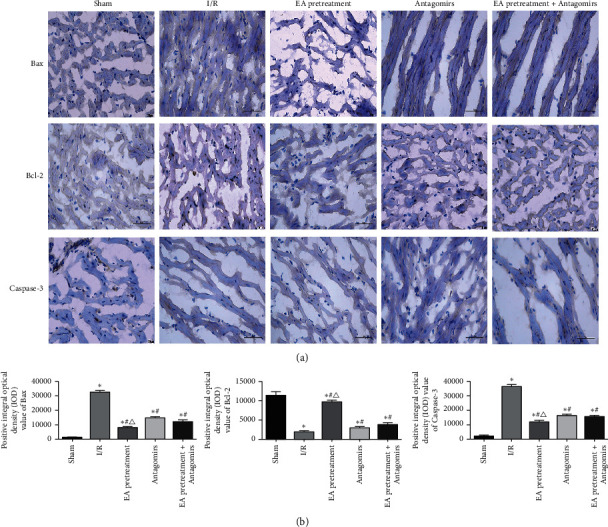
Results of immunohistochemical staining of the myocardium in each group of rats. (a) The myocardial tissue immunohistochemical staining; (b) positive IOD values of Bax, Bcl-2, and Caspase-3 (x ± *s*, *n* = 10). ^*∗*^, *P*<0.05 compared to the Sham group; *P*<0.05 compared to the I/R group; *P*<0.05 compared to the Antagomirs and EA pretreatment + Antagomirs group.

Apoptotic-related molecules (Bax, Bcl-2, and Caspase-3) were observed by staining and IOD values calculated by a fluorescence microscope to investigate the relationship between antimyocardial apoptosis mechanism induced by EA pretreatment and RhoA/p38MAPK pathway and these molecules. Bax (apoptosis-inducing molecule), Bcl-2 (inhibitory apoptosis molecule), and Caspase-3 (apoptosis effector molecule) play a vital role in apoptosis. As shown in Figure 5, Bax, Bcl-2, and Caspase-3 are mainly expressed in the cytoplasm of cardiac myocytes and few in nuclei. Bax and Caspase-3 expressions in the I/R group were significantly higher than the Sham group, while the Bcl-2 expression decreased significantly (*P* < 0.05). Compared with the I/R group, Bax and Bcl-2 levels decreased; however, the Bcl-2 level increased under EA pretreatment intervention (*P* < 0.05). This phenomenon reversed after blocking EA pretreatment with an antagonist.

### 3.5. MKK3, MKK6, p38MAPK, Bax, Bcl-2, and Caspase-3 mRNA Expression

**Figure 6:**
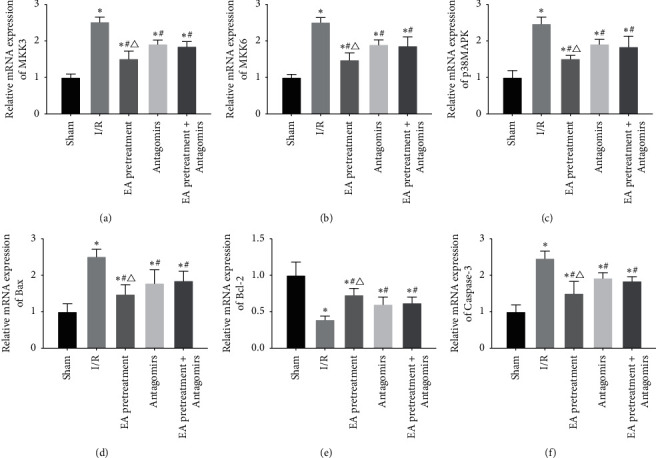
Comparison of the expression levels of MKK3, MKK6, p38MAPK, Bax, Bcl-2, and Caspase-3 mRNA in the myocardial tissue of each group of rats (2^– △△ Ct^, *x* ± *s*, *n* = 10). ^*∗*^, *P* < 0.05 compared to the Sham group; *P* < 0.05 compared to the I/R group; *P* < 0.05 compared to the Antagomirs and EA pretreatment + Antagomirs group.

mRNA expression of MKK3, MKK6, p38MAPK, and apoptosis-associated genes (Bax, Bcl-2, and Caspase-3) was determined by using RT-PCR to confirm whether EA pretreatment affects these molecules via RhoA/p38MAPK pathway, thereby protecting apoptosis of MIRI in rats. As shown in Figure 6, MKK3, MKK6, p38MAPK, Bax, and Caspase-3 mRNA increased significantly compared with the Sham group, while Bcl-2 decreased significantly (*P* < 0.05). Compared with the I/R group, MKK3, MKK6, p38MAPK, Bax, and Caspase-3 mRNA decreased while Bcl-2 increased (*P* < 0.05). After blocking EA pretreatment with an antagonist, this phenomenon reversed.

### 3.6. miR-133a-5p Expression

**Figure 7:**
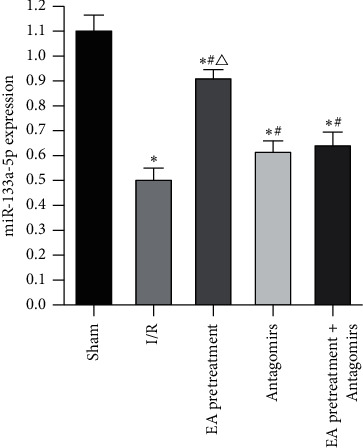
Expression of miRNA-133a-5p in rats from each group. ^*∗*^, *P* < 0.05 compared to the Sham group; *P*<0.05 compared to the I/R group; *P* < 0.05 compared to the Antagomirs and EA pretreatment + Antagomirs group.

Our study used RT-PCR to detect the expression of miR-133a-5p in each group to verify the effect of the RhoA/p38MAPK pathway mediated by changes in miR-133a-5p expression on MIR rats with apoptosis. As shown in Figure 7, miR-133a-5p decreased significantly compared with the Sham group (*P* < 0.05). Compared with the I/R group, miR-133a-5p increased (*P* < 0.05). After blocking EA pretreatment with an antagonist, this phenomenon reversed.

### 3.7. MKK3, MKK6, p38MAPK, Bax, Bcl-2, and Caspase-3 Protein Expression

**Figure 8:**
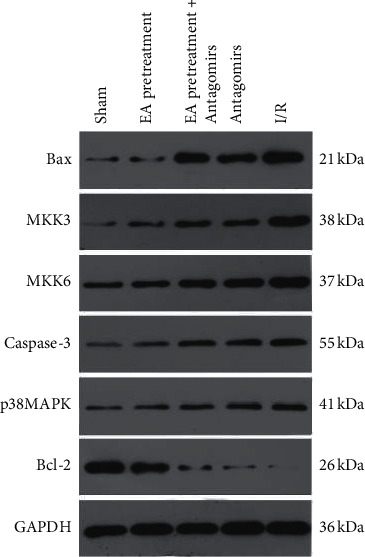
Expression of protein bands in the myocardium of rats in each group.

**Figure 9:**
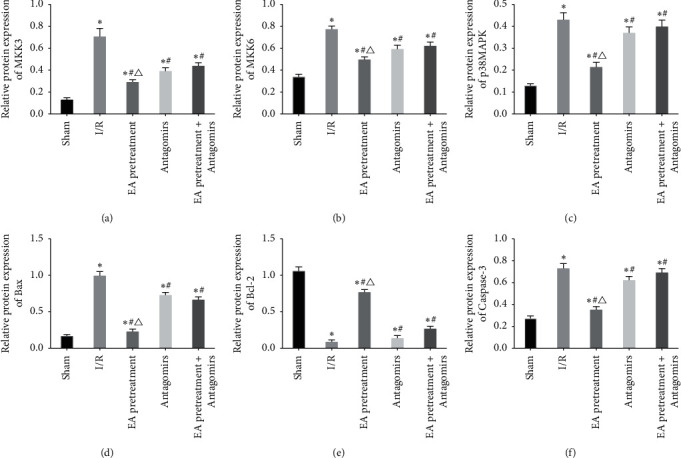
Comparison of protein expression levels of MKK3, MKK6, p38MAPK, Bax, Bcl-2, and Caspase-3 in myocardial tissue of rats in each group (*x* ± *s*, *n* = 10).^*∗*^, *P* < 0.05 when compared to the Sham group; *P* < 0.05 when compared to the I/R group; *P* < 0.05 when compared to the Antagomirs and EA pretreatment + Antagomirs group.

Protein expression of MKK3, MKK6, p38MAPK, and apoptosis-associated protein (Bax, Bcl-2, and Caspase-3) was determined using western blot to verify whether EA pretreatment affects these molecules via RhoA/p38MAPK pathway, thereby protecting apoptosis of MIRI in rats. MKK3, MKK6, p38MAPK, Bax, and Caspase-3 protein increased significantly compared with the Sham group while Bcl-2 decreased (*P* < 0.05) (Figures 8 and 9). Compared with the I/R group, MKK3, MKK6, p38MAPK, Bax, and Caspase-3 protein decreased while Bcl-2 increased (*P* < 0.05). After blocking EA pretreatment with an antagonist, this phenomenon reversed, consistent with the result of RT-PCR.

## 4. Discussion

Myocardial damage during AMI includes MIRI. In recent years, the prognosis of acute myocardial infarction has improved significantly with the introduction of reperfusion methods [15]. MIRI causes myocardial cell necrosis, apoptosis, arrhythmia, and other cardiac dysfunctions. The current treatment for MIRI is divided into nonpharmacological and pharmacological interventions [16]. Many pharmacological and nonpharmacological interventions have been used to reduce fatal reperfusion injury. Reducing reperfusion injury has drawn a lot of attention in treatment methods. However, few interventions have successfully reduced MIRI in recent years, and apoptosis in MIRI has become a key breakthrough.

RhoA is a small guanosine-50-triphosphate binding protein with GDP/GTP binding activity [17], which directly interacts with ROCK and plays a vital role in the pathogenesis of various cardiovascular diseases such as arterial hypertension, atherosclerosis, heart attack, vascular remodeling, myocardial hypertrophy, and MIRI [18]. Studies have shown that [19] the protective effect of RhoA or RhoA effector Rho kinase (ROCK) is related to cardiomyocyte survival and Caspase-3 activation. RhoA protects MIRI after myocardial ischemia. p38MAPK is a subfamily of the MAPK superfamily involved in cell regulation and various stress stimulations [20] and plays a key role in IL-8-induced cell migration. p38MAPK inhibitors cause Rac1 and RhoA proteins to bypass IL-8. Increased expression in treated cells eliminates IL-8-induced p38MAPK activation, forms a signaling pathway with activated RhoA, and promotes mitochondrial autophagy and apoptosis [21, 22]. RhoA, p38MAPK, and signaling protein, focal adhesion kinase (FAK), are involved in Ang II-induced apoptosis of HeLa cells [23]. Thus, the RhoA/p38MAPK pathway has become a research hotspot in the field of apoptosis.

Chinese medicine monomer studies have confirmed [24] that syringic acid (SA) pretreatment significantly downregulates p38MAPK and JNK signaling pathways in H9C2 cardiomyocytes after hypoxia/reoxygenation (H/R) injury. It also significantly reduced the lysis level of Caspases-3 and reduced H/R-induced H9C2 phosphorylation of p38MAPK and JNK in cells, resisted MIRI, and induced cardioprotection. On the contrary, inhibition of p38MAPK increased nuclear concentration and Caspase-3/7 activation, suggesting that p38MAPK has a protective effect. The study showed that activating angiotensin II type-2 receptor (AT2R) can induce FAK and RhoA cell apoptosis and p38MAPK plays a role in prolonging survival. Overexpressed microRNA-485 downregulates the target gene, RhoA, by regulating the RhoA-mediated TGF-*β*-MAPK signaling pathway. RhoA promoted the vitality of rat renal tubular epithelial cells (RTEC) in lupus nephritis (LN) mice and inhibited RTEC cell apoptosis. The key regulatory role of the RhoA/p38MAPK pathway in cell apoptosis has been determined [25].

Apoptosis is a cell death process directed by active genes via two main pathways: exogenous apoptotic and endogenous apoptotic pathway [26]. The former is mainly affected by the Bcl-2 family and the Caspase family. The regulation of apoptosis plays a crucial role in MIRI [27]. Bcl-2 prevents the activation of Bcl-2 and Caspase-3 by forming a heterodimer with Bax [28]. Conversely, MIRI significantly upregulates the expression of Bax in H9C2 cells and downregulates the expression of Bcl-2 and combined with the activation of activated caspases and imbalance of pro/antiapoptotic proteins induce cardiomyocyte apoptosis [29].

The upstream activators of the p38MAPK signaling pathway are MKK3, MKK4, and MKK6, of which MKK3 and MKK6 specifically only activate p38MAPK [30]. In addition, it has been reported that the phosphorylation level of MKK3/6 did not change in the presence of Rho antagonists. In contrast, the phosphorylation level of MKK3/6 and p38MAPK decreased in ROCK antagonist presence, suggesting that Rho is regulated by ROCK phosphorylation levels of MKK3/6 and p38MAPK [31]. Although RhoA and p38MAPK belong to different types of signal transduction proteins, they are closely related to cytoskeleton, connection, differentiation, and apoptosis. All signal pathways involved in p38MAPK may be directly or indirectly associated with RhoA. The establishment of the RhoA/p38MAPK signal transduction pathway provides the basis and method for preventing and treating cardiovascular and cerebrovascular diseases caused by various stress responses [32, 33]. RhoA/ROCK pathway mediates high glucose-induced cardiomyocyte apoptosis via oxidative stress, JNK, and p38MAPK pathways. p38MAPK reduced Bax mRNA expression, Bax/Bcl-2 protein expression ratio, and Caspase-3 protein level and increased Bcl-2 mRNA [34]. Acupuncture PC6 pretreatment also activated the proapoptotic factors (Bax, Bcl-2) and reduced mitochondrial dysfunction [35].

PM RhoA is a potential regulatory target for Pm-miR133 [36]. On the one hand, tanshinone IIA regulates the expression of Caspase-9, CTGF, and RhoA protein levels by upregulating the level of miR-133 and exerting antimyocardial weight on chronic heart failure rats [37]. On the other hand, the lentiviral transmission of miR-133b improved functional recovery after spinal cord injury in mice. This improvement is related to the downregulation of RhoA expression level after six weeks of spinal cord injury. These results support the treatment with targeted miR-133. There is a large amount of miRNA expression in platelets. miRNA affects platelet apoptosis by regulating the expression of Bcl-xL [38]. A significant negative correlation suggested that it may target Bcl-xL (regulatory protein for platelet survival) [39]. In addition, studies have confirmed [40] that the downregulation of miR-133 in gastric cancer is mainly mediated by histone modification in the promoter region, and the restoration of miR-133b/a-3p expression can be directly targeted to antiapoptosis. The molecules, mcl1 and Bcl-xL, inhibit the proliferation of gastric cancer cells and promote apoptosis and then increase the level of miR-133–3p that regulates the antiapoptotic molecules (Caspase-9 and Bcl-xl) to inhibit cell proliferation and promote apoptosis.

In this study, a rat MIR model was established by the physical ligation and push tube method. miR-133a-5p was adjusted to determine whether EA pretreatment would further promote the activation of the RhoA/p38MAPK signaling pathway and subsequently regulate the mRNA and protein levels of Bcl-2, Bax, and Caspase-3, thereby resisting MIRI cell apoptosis. Our previous studies confirmed that the expression of miR-133a-5p decreased significantly after myocardial ischemia in rats and after injection of an antagonist. In this study, the Antagomirs group was established for three days after ischemia-reperfusion (3 mg/L) 80 mg/kg, and mRNA expression was assessed in rats. The study results showed that compared with the I/R group, after tail vein injection of antagomirs, EA pretreatment, EA pre-treatment + antagomirs, and antagomirs significantly downregulated positive IOD and MKK3, MKK6, p38MAPK, Bax, and Caspase-3 protein and mRNA expression. Besides, the protein and mRNA expression levels of Bcl-2 and positive IOD values were increased (*P* < 0.05). However, the EA pretreatment group had the most significant effect. There was no significant difference between the EA pretreatment + Antagomirs group and the Antagomirs group (*P* > 0.05). The efficacy of the EA pretreatment group was significantly better. The results showed that EA pretreatment significantly reduced the damage of the I/R to cardiac function. The specific mechanism of EA pretreatment of ”point matching of specimen” involves the downregulation of MKK3, MKK6, and p38MAPK via the RhoA/p38MAPK pathway, thereby reducing the expression of the proapoptotic molecule (Bax) and the apoptotic effector (Caspase-3) and upregulating the antiapoptotic factor (Bcl-2) simultaneously, thus protecting the apoptosis of MIRI in rats. In this study, the expression of TUNELpositive cells in the Sham group was slightly higher, which may be due to the following: on the one hand, the heart exposure time after thoracotomy in the Sham group was the same as that in the I/R group. Because the rats in the study were not connected to a ventilator, pneumothorax, collapsed lungs, and damaged cardiomyocytes occurred when the chest was opened, leading to apoptosis. Compared with the I/R operation connected with an animal ventilator, this model has a slightly greater damage to the heart of rats, but does not affect the results and conclusions of the study. On the other hand, the whole modeling process lasted for 60 min (ischemia for 20 minutes, reperfusion 40 min), and the exposure time in the Sham group was the same as that in the I/R group. During this period, external environmental stimuli (such as 4% paraformaldehyde in the indoor air of the laboratory and the emission of chloral hydrate) caused partial damage to the heart and led to apoptosis. In this experiment, although there is a particular difference between the Antagomirs group and the I/R group, the difference is higher than the EA pretreatment and the EA pretreatment + Antagomirs group. EA pretreatment protective effect of ”point matching of specimen” on MIRI may also be caused by multiple targets and diverse pathways apart from the RhoA/p38MAPK pathway. Moreover, we need to study further the clinical application of “point matching of specimen” and EA preconditioning in myocardial injury caused by diseases such as AMI and other related heart injuries.

## Data Availability

The data used to support the findings of this study are available from the corresponding author upon request.

## References

[1] Mori K., Lee H. T., Rapoport D. (2005). Endocytic delivery of lipoca linsiderophoreiron complex rescues the kidney from ischemia-reperfusion injury. *Journal of Clinical Investigation*.

[2] Hashmi S., Al-Salam S. (2015). Acute myocardial infarction and myocardial ischemia- reperfusion injury: a comparison. *International Journal of Clinical & Experimental*.

[3] Jurlander B., Clemmensen P., Ohman E. M., Christenson R., Wagner G. S., Grande P. (1996). Serum myoglobin for the early non-invasive detection of coronary reperfusion in patients with acute myocardial infarction. *European Heart Journal*.

[4] Hentia C., Rizzato A., Camporesi E. (2018). An overview of protective strategies against ischemia/reperfusion injury: The role of hyperbaric oxygen preconditioning. *Brain and Behavior*.

[5] Chen S., Wu S., Han Y.-L. (2017). Effects of Electroacupuncture at neiguan point on cardiac function, myocardial injury markers and morphology in rats with myocardial ischemia reperfusion model. *Lishizhen Medicine and Materia Medica Research*.

[6] Chen S., Han Y.-L., Wu S. (2017). Protective effect of acupuncture “Neiguan” preconditioning on myocardial ischemia-reperfusion in rats and its mechanism. *Acta Medicine Universitis Scientiae et Technologiae Huazhong*.

[7] Chen S., Chen Q., Wu S., Wang H., Liang F.-X., Huang W. (2018). Effects of electroacupuncture pretreatment on specimen acupoints on myocardial mitochondrial respiratory function in rats with chronic myocardial ischemia. *Liaoning Journal of Traditional Chinese Medicine*.

[8] Chen S., Wu S., Wang H., Liang F.-X. (2017). Research progress on P38 signal transduction pathway and disease spectrum. *Journal of Hubei University of Traditional Chinese Medicine*.

[9] Huang Y., Chen J.-B., Yang B., Shen H., Liang J.-J., Luo Q. (2014). RhoA/ROCK pathway regulates hypoxia-induced myocardial cell apoptosis. *Asian pacific Journal of Tropical Medicine*.

[10] Zhou H., Sun Y.-H., Zhang L.-H., Kang W.-Y., Li N., Li Y.-J. (2018). The RhoA/ROCK pathway mediates high glucose-induced cardiomyocyte apoptosis via oxidative stress, JNK, and p38MAPK pa thways. *Diabetes/metabolism Research & Reviews*.

[11] Surma M., Wei L., Shi J. (2011). Rho kinase as a therapeutic target in cardiovascular disease. *Future Cardiology*.

[12] Lu J.-D. (2016). Effect of “specimen with acupoints” on apoptotic miRNA in myocardial ischemic rats and its regulatory mechanism. *Hubei University of Traditional Chinese Medicine*.

[13] Wang M.-X., Liao J.-H., Ma D., Hu D.-H., Wang C.-G., Liang J. (2013). Establishment of rat in vivo myocardial ischemia-reperfusion model. *Clinical Medicine & Engineering*.

[14] Guang W. (1993). Research on acupuncture points of experimental animals passed identification. *Chinese Acupuncture and Moxibustion*.

[15] Werf F., Van D. (1993). Reperfusion treatment in acute myocardial infarction in elderly patients. *Kardiologia polska*.

[16] Luo S.-Y., Chen S., Qin Y.-D., Chen Z.-W. (2016). Urotensin-II receptor antagonist SB-710411 protects rat heart against ischemia-reperfusion injury via RhoA/ROCK pathway. *PLoS One*.

[17] Aspenström P. (2019). The intrinsic GDP/GTP exchange activities of Cdc42 and Rac1 are critical determinants for their specific effects on mobilization of the actin filament system. *Cells*.

[18] Liu Y., Minze L.-J., Mumma L., Li X.-C., Ghobrial R.-M., Kloc M. (2016). Mouse macrophage polarity and ROCK1 activity depend on RhoA and non-apoptotic Caspase 3. *Experimental Cell Research*.

[19] Hannan J. L., Matsui H., Sopko N. A. (2016). Caspase-3 dependent nitrergic neuronal apoptosis following cavernous nerve injury is mediated via RhoA and ROCK activation in major pelvic ganglion. *Scientific Reports*.

[20] Deng R., Li F., Wu H. (2018). Anti-inflammatory mechanism of geniposide: inhibiting the hyperpermeability of fibroblast-like synoviocytes via the RhoA/p38MAPK/NF-*κ*B/F-Actin signal pathway. *Frontiers in Pharmacology*.

[21] Lai Y., Liu X.-H., Zeng Y., Zhang Y., Shen Y., Liu Y. (2012). Interleukin-8 induces the endothelial cell migration through the Rac1/RhoA-p38MAPK pathway. *European Review for Medical and Pharmacological Sciences*.

[22] Lai Y., Shen Y., Liu X.-H., Zhang Y., Zeng Y., Liu Y.-F. (2011). Interleukin-8 induces the endothelial cell migration through the activation of phosphoinositide 3-kinase-Rac1/RhoA pathway. *International Journal of Biological Ences*.

[23] Ding S.-K., Wang L.-X., Guo L.-S. (2017). Syringic acid inhibits apoptos is pathways via downregulation of p38MAPK and JNK signaling pathways in H9c2 cardiomyocytes following hypoxia/reoxygenation injury. *Molecular Medicine Reports*.

[24] Kamel W., Sugihara E., Nobusue H. (2016). Simvastatin-induced apoptosis in osteosarcoma cells: a key role of RhoA-AMPK-p38 MAPK signaling in antitumor activity. *Molecular Cancer Therapeutics*.

[25] Tian Y., Han Y.-X., Guo H.-F. (1998). Upregulated microRNA-485 suppresses apoptosis of renal tubular epithelial cells in mice with lupus nephritis via regulating the TGF-*β*-MAPK signaling pathway by inhibiting RhoA expression. *Journal of Cellular Biochemistry*.

[26] Sandro A., Angélica G.-C., Cano M., Mario F. M., Ayala A. (2019). Advantages and disadvantages of apoptosis in the aging process. *Annals of the New York Academy of Sciences*.

[27] Alabsi A. M., Kai L. L., Paterson I. C., Ali-Saeed R., Muharram B. A. (2016). Cell cycle arrest and apoptosis induction via modulation of mitochondrial integrity by bcl-2 family members and caspase dependence in dracaena cinnabari-treated h400 human oral squamous cell carcinoma. *BioMed Research International*.

[28] Shen J., Wu Z., Wang M. H. (2015). Epigenetic silencing of miR-490-3p reactivates the chromatin remodeler SMARCD1 to promote Helicobacter pylori-induced gastric carcinogenesis. *Cancer Research*.

[29] Gottlieb R. A., Engler R. L. (2010). Apoptosis in myocardial ischemia-reperfusion. *Annals of the New York Academy of Sciences*.

[30] Stramucci L., Pranteda A., Bossi G. (2018). Insights of crosstalk between p53 protein and the MKK3/MKK6/p38 MAPK signaling pathway in cancer. *Cancers*.

[31] Ali T. K., Al-Gayyar M. M. H., Matragoon S. (2011). Diabetes-induced peroxynitrite impairs the balance of pro-nerve growth factor and nerve growth factor, and causes neurovascular injury. *Diabetologia*.

[32] Kwak H. H., Kim I. R., Kim H. J., Park B. S., Yu S. B. (2016). *α*-Mangostin induces apoptosis and cell cycle arrest in oral squamous cell carcinoma cell. *Evidence Based Complement Alternative Medicine*.

[33] Manzur M. J., Aguilera M. O., Kotler M. L., Berón W., Ciuffo G. M. (2018). Focal adhesion kinase, RhoA, and p38 mitogen-activated protein kinase modulates apoptosis mediated by angiotensin II AT receptors. *Journal of Cellular Biochemistry*.

[34] Chen Y.-J., Liu W.-H., Kao P.-H., Wang J.-J., Chang L.-S. (2010). Involvement of p38 MAPK- and JNK-modulated expression of Bcl-2 and Bax in Naja nigricollis CMS-9-induced apoptosis of human leukemia K562 cells. *Toxicon*.

[35] Chen S. (2017). *Effect of Electroacupuncture Pretreatment at “neiguan” on the p38MAPK Signaling Pathway in Rats with Myocardial Ischemia-Reperfusion Injury*.

[36] Zheng Z., Huang R., Tian R., Jiao Y., Du X. (2016). Pm-miR-133 hosting in one potential lncRNA regulates RhoA expression in pearl oyster Pinctada martensii. *Gene*.

[37] Feng J., Chen H.-W., Li S.-S. (2016). Tanshinone II A for chronic heart failure and its mechanism to examine the effects of myocardial remodeling. *Journal of Emergency in Traditional Chinese Medicine*.

[38] Adams C. M., Hiebert S. W., Eischen C. M. (2016). Myc induces miRNA-mediated apoptosis in response to HDAC inhibition in hematologic malignancies. *Cancer Research the Official Organ of the American*.

[39] Yu S.-F., Li Q., Ye Y.-Y., Wu L.-F., Xia K.-D., Xie Z.-T. (2015). Expression of miRNAs targeting bcl-xl in platelet storage and its effect on platelet apoptosis. *Chinese Journal of Health Laboratory Technology*.

[40] Theis T., Yoo M., Park C. S. (2017). Lentiviral delivery of miR-133b improves functional recovery after spinal cord injury in mice. *Molecular Neurobiology*.

